# Can Muscle Building Supplements Increase Testicular Cancer Risk?

**DOI:** 10.3389/fnut.2022.778426

**Published:** 2022-01-28

**Authors:** Kevin G. Girardi, Tongzhang Zheng, Yong Zhu

**Affiliations:** ^1^Department of Environmental Health Sciences, Yale University School of Public Health, New Haven, CT, United States; ^2^Brown University School of Public Health, Providence, RI, United States

**Keywords:** muscle building supplements, cancer risk, testicular cancer, hormone and prohormone substances, chemical and environmental contaminants

## Introduction

Testicular germ cell cancer (TGCC) is the most common cancer type among men aged 14–44 (1). It is widely known that genetic factors play a major role in testicular cancer risk: male individuals who have a brother with a history of testicular cancer have an 8–12-fold increased risk of developing the disease (2), and there is also a marked increase in risk in association with a personal history of cryptorchidism (3). However, genetic factors including cryptorchidism cannot explain why rates of TGCC have grown from 3.73 per 100,000 in 1975 to 6.31 per 100,000 in 2017 (4), and there is increasing evidence suggesting that environmental exposures, especially those during adolescence, may increase testicular cancer risk by hormone disruption resulting in modified testicular function and damaging and modifying the function of susceptibility genes (5).

A growing public health concern, especially among athletes, is the prevalent use of dietary supplements for muscle building effects in an attempt to enhance performance (6). One study found 87.5% of elite athletes, defined as those training in various sports at a state-based institute, took dietary supplements (7). The global dietary supplement industry was estimated at US$123.28 billion in 2019 and is rapidly growing (8). In addition to increasing athletic performance, another possible explanation for the growing use of muscle building supplements (MBS) is societal pressure for teenage boys and young men to conform to an idealized muscular body (9).

Questions about the health risks of MBS have long been asked, but studies have mostly focused on short term outcomes. One of the few long-term health risks, and the only carcinogenic risk to our knowledge, that has been identified in association with MBS use is TGCC. It was reported that about 20 percent of TGCC patients were taking performance enhancing substances at the time of diagnosis (10). A follow up population case-control study of Connecticut and Massachusetts TGCC patients identified MBS use as a potential risk factor associated with TGCC (odds ratio = 1.65), especially among those who used MBS prior to age 25 (odds ratio = 2.21) (11).

When examining MBS, it can be very difficult to classify dietary supplements as specifically for muscle building, as there are many products better classified as multipurpose “stacks” to indicate general workout supplements that are designed for energy, muscle building, and/or weight loss. A specific type of stack, multi-ingredient pre-workout supplements (MIPS), have become very popular in recent years, and often contain banned substances (12).

The FDA defines dietary supplements as products taken orally that contain a “dietary ingredient.” Whether MBS necessarily need to meet this definition has not been clearly established, so the line between anabolic androgenic steroids (AAS) and MBS becomes difficult to define, especially considering that some MBS are often adulterated with AAS. All published papers to our knowledge only included products ingested orally and referred to products containing dietary ingredients, indicating that MBS are simply dietary supplements. However, the two population studies, Chang et al. and Li et al., considered androstenedione use as MBS use (10, 11). While androstenedione is in fact not a dietary ingredient but rather an anabolic steroid, Chang et al. refer to “performance enhancing supplements” rather than “muscle building supplements” which could explain its inclusion (10). Also, androstenedione was originally considered a dietary supplement and then was reclassified as an anabolic steroid in 2004. Li et al. state that androstenedione showed up in their analysis of 30 MBS, so it may have appeared alongside dietary ingredients or as a contaminant of certain MBS (11). While the roles of androstenedione and other AAS in testicular cancer are certainly worth investigating, muscle building dietary supplements users make up a much larger proportion of MBS users than do people who take muscle building products without dietary ingredients.

Currently, the FDA does not regulate the supplement industry for safety until after the products are on the market. These products are sold legally throughout the United States. The industry is massive and growing fast, with sales in “sports nutrition products” expected to reach 44.3 billion dollars by 2021, making it unrealistic to test a significant proportion of products on the market (13). Potentially harmful ingredients found in muscle building supplements are wide ranging from impurities, to prohormones, to banned substances, to pharmaceuticals. Furthermore, many of these supplements are tainted substances that have compounds that are not listed on the label, likely intentionally to try to enhance their effects (14). Others have environmental contaminants like bisphenol A (BPA) and lead. Some have substances that are legal and listed on the label but are still possibly harmful and/or unstudied.

## Commonly Used Supplements

Protein, creatine, amino acids, energy boosters, diuretics, and weight loss supplements are among the most commonly used supplements, according to a national study of NCAA male athletes (15). The case-control study by Li et al. states that their assessment considered 30 different powders and pills reported by patients—with creatine, protein, and androstenedione or its booster as the main ingredients) (11).

Overall, pure creatine is regarded as safe for use within the research community (16). We are unaware of any studies definitively linking protein supplementation to carcinogenic effects. Androstenedione has also recently been categorized as a schedule III-controlled substance (17), which hopefully has decreased its use.

Since little evidence exists attributing the most used MBS, creatine and protein, as carcinogenic in their purified forms, it is worth considering that the association reported by Li et al. could be due to other compounds that are commonly found in products that are labeled as creatine or protein, or the possibility that people taking creatine or protein are also taking other workout supplements or anabolic steroids. Table 1 summarizes some MBS ingredients, contaminants, and adulterants that have been identified as potential risk for carcinogenic effects, hormone disruption, or testicular damage.

**Table 1:** MBS ingredients identified as potential risk for carcinogenic effects, hormone disruption, or testicular damage.

**Compound name**	**Purpose**	**Information and potential risk**
**Ingredients**		
Lorcaserin	Diuretic effects	Found in weight loss/body building supplements. Carcinogenic warning from FDA (25).
Phenolphthalein	Laxative effects	Found in weight loss/body building supplements. Carcinogenic risk from FDA, found to be genotoxic (26).
**Environmental contaminants**		
BPA	Contaminant	55% of top protein powders tested by the clean label project had BPA (19). BPA is carcinogenic, and specifically been linked to testicular tumorigenesis in mice (27, 28).
Lead	Contaminant	75% of the top protein supplements tested by clean label project had detectable levels of lead (19). Shown to raise testosterone in males (21).
Cadmium	Contaminant	74% of protein powders contained cadmium (19). Shown to raise testosterone in males (21).
Arsenic	Contaminant	Found in protein powders (29). Caused testicular damage in animal studies (30).
**Prohormone substances**		
AAS found in MBS
Prostanozolol 19-norandrosterone Methasteron Dehydroepiandrosterone	Increase testosterone or other similar androgen levels.	Two of the most common types of AAS are Nandrolone and Stanozolol have been shown to enhance Leydig cell proliferation and increase tumor development risk (shown in animal studies only so far) (24). All AAS are listed as probable carcinogens by the International Agency for Research on Cancer (IARC) (20). Androstenedione is a precursor to testosterone that specifically has been shown to cause testicular atrophy in men (17).
(DHEA)		
Methandienone		
Desoxymethyltestosterone		
Nandrolone		
4-chlorodehydromethyltestosterone		
Stanozolol		
Androstenedione		
**Selective Androgen Receptor Modulators (SARMs)**		
Ostarine Andarine	Designed to have an anabolic effect only in particular tissues (Muscle and Bone).	FDA statement on SARMs “Body-building products that contain selective androgen receptor modulators, or SARMs, have not been approved by the FDA and are associated with serious safety concerns, including potential to increase the risk of heart attack or stroke and life-threatening reactions like liver damage.” The FDA also notes long-term effects on the body are unknown (31).

## Contaminants in Supplements

Since the United States FDA does not regulate supplements prior to their availability on the market, much of the information available regarding environmental contaminants comes from the private sector. Entities such as Labdoor, Consumerlabs, and The Clean Label Project have taken up the task of testing supplements for contaminants.

Heavy metals that have been found in MBS include cadmium and lead (18, 19). Cadmium is a known endocrine disruptor and testicular injury can occur at relatively low levels of exposure, although it has only been directly linked to TGCC in animal studies to far (5). Lead is listed in The International Agency for Research on Cancer (IARC) as a group 2A probable carcinogen (20), and has also been linked to hormonal effects including elevated testosterone levels (21), and specific effects in the testicles (22). Whether Lead, Cadmium, or BPA appear in high enough concentrations within MBS to cause health effects remains a question.

**Figure 1:**
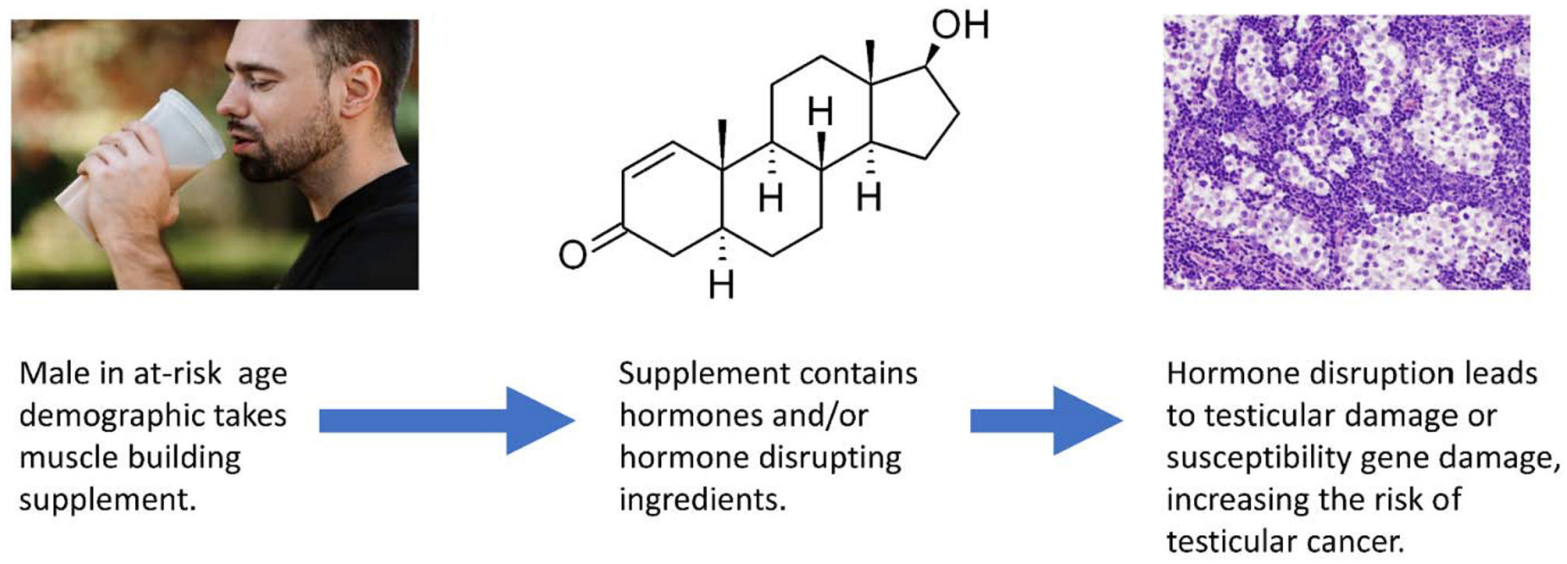
An illustration of MBS increasing testicular cancer risk by hormone disruption.

A critical response to Li et al. case control study was the suggestion that many of the users of MBS are also using anabolic androgenic steroids (AAS). MBS are often adulterated or contaminated with AAS (23). Two of the most common anabolic steroids, nandrolone and stanozolol, have been shown to enhance testicular Leydig cell proliferation and increase tumor development risk in animal models (24). Anabolic steroids are classified under group 2A as a probable carcinogen by the IARC in relation to multiple hormone related cancers, although not yet testicular cancer (20). It is certainly possible that elevated probability of concomitant use between MBS and anabolic steroids is responsible for some of the risk observed. Figure 1 illustrates that MBS may increase testicular cancer risk by disrupting hormone levels.

## Future Directions

Given that limited studies have been conducted to examine the role of MBS in cancer development, more population-based investigations are needed to further confirm the previously observed associations. Participant use habits are often inconsistent over time as subjects commonly switch brands, type, and doses of MBS among other factors for a variety of reasons, which makes it difficult to gathering coherent population data of MBS. In future study, it is important to have a detailed assessment that considers the potential instabilities in each exposure assessment. The use of multiple substances and use at younger ages are factors of particular interest as well, as these have been identified as potentially stronger risk factors by Li et al. (11), it is also of interest to gather population data on a potential association of MBS use with other cancer that are affected by hormonal pathways, including other types of testicular cancers. Moreover, toxicological studies are needed to interrogate adverse health effects of more ingredients and contaminants in MBS, which will help understand potential risks of using MBS and facilitate monitoring regulation of the ingredients in MBS to minimize exposure to carcinogenic compounds.

## Conclusions

MBS may be a growing public health concern as their use continues to increase, especially among athletes and adolescences (13). They often contain harmful ingredients including those with carcinogenic potential, and MBS related chemical exposures can disrupt hormone pathways and may be contributing to testicular cancers. In addition to continued study of population data, we believe that the FDA should be regularly testing products in an over 40-billion-dollar industry that so often puts adulterated and contaminated products on retail shelves (13). It is also important to better educate and affect teen attitudes about the use of MBS, and to consider making them less available to younger people.

## Author Contributions

TZ and YZ: conception and design. KG and YZ: writing, review, and revision of the manuscript. All authors have read and approved the manuscript.

## Funding

This work was supported by funds from Yale University. KG's internship was supported by NIEHS training grant R25ES029052.

## Conflict of Interest

The authors declare that the research was conducted in the absence of any commercial or financial relationships that could be construed as a potential conflict of interest.

## Publisher's Note

All claims expressed in this article are solely those of the authors and do not necessarily represent those of their affiliated organizations, or those of the publisher, the editors and the reviewers. Any product that may be evaluated in this article, or claim that may be made by its manufacturer, is not guaranteed or endorsed by the publisher.
